# Metabolic and Blood Pressure Effects of Walnut Supplementation in a Mouse Model of the Metabolic Syndrome

**DOI:** 10.3390/nu9070722

**Published:** 2017-07-07

**Authors:** Nicola J. A. Scott, Leigh J. Ellmers, Anna P. Pilbrow, Lotte Thomsen, Arthur Mark Richards, Chris M. Frampton, Vicky A. Cameron

**Affiliations:** 1 Christchurch Heart Institute, University of Otago—Christchurch, P.O. Box 4345, Christchurch 8140, New Zealand; nicola.scott@otago.ac.nz (N.J.A.S.); Leigh.ellmers@otago.ac.nz (L.J.E.); anna.pilbrow@otago.ac.nz (A.P.P.); lottekthomsen@icloud.com (L.T.); arthur_mark_richards@nuhs.edu.sg (A.M.R.); Statistecol@xtra.co.nz (C.M.F.); 2 Cardiovascular Research Institute, National University of Singapore, 1E Kent Ridge Road, Singapore 119228, Singapore

**Keywords:** metabolic syndrome, walnuts, glucose tolerance, cholesterol, gene expression

## Abstract

There is extensive evidence that walnut consumption is protective against cardiovascular disease and diabetes in the healthy population, but the beneficial effects of walnut consumption in individuals with the metabolic syndrome (MetS) remain uncertain. We compared a range of cardio-metabolic traits and related tissue gene expression associated with 21 weeks of dietary walnut supplementation in a mouse model of MetS (MetS-Tg) and wild-type (WT) mice (*n* = 10 per genotype per diet, equal males and females). Compared to standard diet, walnuts did not significantly alter food consumption or body weight trajectory of either MetS-Tg or WT mice. In MetS-Tg mice, walnuts were associated with reductions in oral glucose area under the curve (gAUC, standard diet 1455 ± 54, walnut 1146 ± 91, *p* = 0.006) and mean arterial blood pressure (MAP, standard diet 100.6 ± 1.9, walnut 73.2 ± 1.8 mmHg, *p* < 0.001), with neutral effects on gAUC and MAP in WT mice. However, in MetS-Tg mice, walnuts were also associated with trends for higher plasma cholesterol (standard diet 4.73 ± 0.18, walnut 7.03 ± 1.99 mmol/L, *p* = 0.140) and triglyceride levels (standard diet 2.4 ± 0.5, walnut 5.4 ± 1.6 mmol/L, *p* = 0.061), despite lowering cholesterol and having no effect on triglycerides in WT mice. Moreover, in both MetS-Tg and WT mice, walnuts were associated with significantly increased liver expression of genes associated with metabolism (*Fabp1*, *Insr*), cell stress (*Atf6*, *Ddit3*, *Eif2ak3*), fibrosis (*Hgf*, *Sp1*, *Timp1*) and inflammation (*Tnf*, *Ptpn22*, *Pparg*). In conclusion, dietary walnuts were associated with modest favourable effects in WT mice, but a combination of beneficial and adverse effects in MetS-Tg mice, and up-regulation of hepatic pro-fibrotic and pro-inflammatory genes in both mouse strains.

## 1. Introduction

The metabolic syndrome (MetS) is a pre-diabetic state associated with both significantly increased cardiovascular risk and poor prognosis in established heart disease [1]. Three or more of the following five risk factors are required for diagnosis: abdominal obesity, elevated fasting triglycerides, reduced HDL cholesterol, high blood pressure or elevated fasting plasma glucose [1]. The MetS is associated with a two-fold increased risk of coronary heart disease (CHD) [2], a 3- to 4-fold increased risk of mortality due to CHD [3] and a 6-fold increased risk of developing type-2 diabetes (DM2) [4]. Globally, an estimated 318 million adults have impaired glucose tolerance, and numerous lifestyle and pharmacological interventions have been trialed to prevent progression to DM2 in high-risk individuals [5].

There is extensive evidence from large prospective studies in the healthy population that nut consumption is protective against cardiovascular disease (CVD) and beneficial for diabetes prevention [6,7,8,9,10]. Subjects who consumed nuts two or more times per week had a 47% reduced risk of sudden cardiac death versus those who rarely or never consumed nuts [11]. The PREvención con DIeta MEDiterránea (PREDIMED) randomized trial assessed long-term effects of a Mediterranean diet incorporating either olive oil or nuts [12], and demonstrated reduced all-cause mortality associated with greater α-linolenic acid intake, principally from intake of walnuts and olive oil [13]. Despite nuts being energy dense food, data collected from over 12,000 participants showed a lower body mass index (BMI) in those subjects who consumed nuts compared to those who never ate nuts (23.8 versus 25.0 kg/m^2^) [14]. Intervention studies show that including nuts, especially walnuts, in an energy-restricted diet can promote weight loss or at least limit weight gain [15,16]. There is also evidence for beneficial effects of nuts on insulin sensitivity; an inverse association between nut consumption and risk of developing DM2 has been reported [9,17]. 

The effects of walnut consumption may depend on the metabolic state, with a neutral effect on insulin sensitivity reported in healthy humans [10], but reduced insulin resistance described in patients with MetS [18] and in overweight or obese women [15]. The reported effects of walnuts on lipids have been mixed and may also depend on metabolic health. Reductions in total and LDL-cholesterol have been observed in healthy participants [6,10,15,19] in one study of adults at-risk for diabetes [16], and a recent randomized control trial of walnut oil in hyperlipidemic DM2 patients [20]. However, elsewhere, a lack of beneficial effects on lipids in MetS has been reported [18]. Reports on the possible effects of dietary walnuts on blood pressure have been similarly disparate, with either lower [18] or no change in blood pressure [16] documented in studies conducted in patients with the MetS. Hence, in patients with existing metabolic disorders, the evidence for global beneficial effects of dietary walnut supplementation remains unclear.

We have previously established a mouse model [21] that displays the key characteristics of human MetS, by crossing two mouse strains, Aromatase-deficient (ARKO) mice with Apolipoprotein E-deficient (ApoE^−/−^) mice. Each of the parent strains carry some of the characteristics of the syndrome, and the double knockout mice exhibit the diverse features fulfilling the criteria of the metabolic syndrome, including central obesity, progressive hypertension, an adverse serum lipid profile, fatty liver, glucose intolerance, insulin resistance and evidence of an inflammatory state [21]. To clarify contrasting previous information on the effects of walnut consumption in individuals with MetS, we have documented the response to walnut supplementation on a wide range of cardio-metabolic traits and related tissue gene expression, comparing healthy mice to the mouse model of metabolic syndrome (MetS-Tg).

## 2. Materials and Methods

### 2.1. Animals and Genotyping

The MetS-Tg mouse strain was generated by crossing two transgenic mouse strains, Aromatase-deficient (ARKO) mice with Apolipoprotein E-deficient (ApoE^−/−^) mice, as described previously [21]. Wild-type and MetS-Tg mice were from a C57Bl/6 background, with the WT mice originating from first generation progeny of the dual-heterozygote-deficient cross from which the MetS-Tg were also derived. Genotyping of MetS-Tg mice born into the Christchurch Heart Institute (CHI) colony was performed as previously described [21], using specific polymerase chain reaction (PCR)-based genotyping assays on genomic DNA extracted from 2 mm of tail tissue collected under anesthesia from 4 week old mice using a DirectPCR^®^ DNA extraction system (Viagen Biotech Ltd., Los Angeles, CA, USA). Mice were housed in individually ventilated cages (3 mice/cage) and maintained a steady temperature (20–24 °C) and humidity (40%) with a standard 12-h light/dark cycle. Experimental protocols were approved by the Animal Ethics Committee of the University of Otago, (Christchurch Approval Reference C809).

### 2.2. Experimental Diets

Three-month old mice received the dietary intervention for 21 weeks. MetS-Tg and WT mice (*n* = 10 per genotype per diet, equal males and females) were placed on a control diet of standard rodent standard diet (Meat free AIN93G Rodent Diet, Specialty Feeds, Australia, Supplementary Table S1), or the experimental diet of standard rodent standard diet reconstituted to include ground walnuts (0.0125 grams per mouse per day, 0.3% standard diet content by weight, for estimated composition, see Supplementary Table S1). This is equivalent to the same human dietary walnut supplement recommended by the US Food and Drug Administration [22] (43 g/day), adjusted for the mean body weights of 3-month old mice (20.4 g) relative to humans (70 kg). The human equivalent dose (HED) has been calculated according the method of Reigner and Blesch [23] (HED in mg/kg) = Animal dose (mg/kg) × Animal K_m_ (in a mouse this equals 3) ÷ Human K_m_ (37) = 0.496 mg of walnuts per day/kg body weight). For the walnut supplemented diet, English Walnuts (*Juglans regia* Chandler variety, sourced from Tasti™, Auckland, New Zealand) with the skin on, were ground to a course powder and then reformed into pellets with the addition of sterile water. The walnut diet was isocaloric with the standard diet but with a slightly higher polyunsaturated fat content, due to a combination of marginally higher levels of palmitic, oleic, linoleic and α linolenic acids. The dietary regime of 21 weeks covers the period between 3–6 months of age when MetS-Tg exhibit the full metabolic syndrome phenotype, and this period is not confounded by either puberty (average 42 days in a mouse) or developing senescence (from 10 months in mice) [24]. Food intake for each cage of animals was evaluated over 3 randomly chosen, 24-h periods between weeks 4–21 of the dietary intervention. Briefly, at 9 am, the mice were removed to a clean cage and a known volume of food was placed in the cradle, the mice were replaced and 24 h later the weight of remaining food was recorded.

### 2.3. Physiological Characterization

Conscious animals were weighed and their blood pressure measured by a non-invasive, computerized tail-cuff system (ADInstruments, Dunedin, New Zealand) prior to commencement of dietary intervention and weekly for the duration of the study period, as previously described [21]. The mice were familiarized to the procedure by being placed briefly in the restrainer and tail cuff system over a period of 7 days, after which MAP measurements were made for each animal (mean of at least ten recordings). 

### 2.4. Glucose and Lipids 

Oral glucose response was determined prior to starting the dietary intervention and repeated prior to sacrifice, after 20 weeks on the diet. After a 14-h fast, mice underwent an oGTT following the mouse protocol of Akagiri et al. [25], with 2 g/kg of glucose administered via oral gavage. Peripheral plasma glucose levels were determined from tail-vein blood samples using an automated glucometer (ACCU-CHEK^®^ Advantage II; Roche Diagnostics GmBH, (Mannheim, Germany) prior to oral glucose administration and 15, 30, 60, 90 and 120 min post glucose gavage. At sacrifice, blood was collected via cardiac puncture and plasma (*n* = 6 mice/group) and serum (*n* = 4 mice/group) samples stored at −80 °C until analyzed. Plasma cholesterol, triglyceride and creatinine levels were measured by quantitative colorimetric assays (Bioassay Systems, Hayward, CA, USA) according to the manufacturer’s instructions. The detection limits were 0.13 mmol/L (5 mg/dL), 0.01 mmol/L (0.88 mg/dL) and 8.8 μmol/L (0.1 mg/dL), respectively.

### 2.5. Tissue Histology 

At the completion of study, after measuring body weight and blood pressure, mice were euthanized with an anesthetic overdose (Isoflurane™) before cervical dislocation. Heart, kidney and liver tissues were rapidly excised, and cardiac atria, ventricles, left kidney, and median lobe of the liver were immediately snap frozen in liquid nitrogen for RNA isolation. The remaining liver tissue, along with a randomly-selected subset of excised hearts (*n* = 3), were emersion fixed in 10% neutral buffered formalin for 24 h, and paraffin embedded for histological analysis. Histology was performed on 5-μm paraffin sections (Leica Microsystems, Wetzlar, Germany) stained with Massons Trichrome and examined under bright-field illumination (Olympus BX50, Hamburg, Germany).

### 2.6. RNA Isolation, cDNA Synthesis and Quantitative Real-Time PCR (RT-qPCR)

Total RNA was isolated from frozen tissue (liver, 450 mg; kidney, 200 mg; cardiac ventricle, 125 mg) by automated grinding in a Retsch (Haan, Germany) MM301 tissue mill (30 Hz, 10 min) in TRIzol™ (Invitrogen, Carlsbad, CA, USA). RNA was purified with RNeasy Midi Columns (Qiagen, Hilden, Germany). RNA concentration and integrity, measured on a 2200 TapeStation (Agilent Technologies, Waldbronn, Germany), ranged from 350–1800 ng/μL and 5.9–8.8 RNA integrity number equivalents (RIN^e^), respectively, and did not differ by genotype or diet. cDNA synthesis was performed in duplicate from 2.5 μg of DNAse I-treated (Roche, Mannheim, Germany) total RNA using SuperScript^®^ IV Reverse Transcriptase (Life Technologies, Carlsbad, CA, USA), followed by treatment with RNase H (Life Technologies).

Expression of genes previously reported to be involved in atherosclerosis, diabetes, inflammation and fatty liver (Table 1) were investigated using Taqman gene expression assays (Life Technologies) run in triplicate (10 μL) on a Lightcycler 480 Real-Time PCR System (Roche Diagnostics, Indianapolis, IN, USA). Gene expression levels (threshold cycle, Ct, values) were calculated with Lightcycler software, release 1.5.1 (Roche). Expression levels were converted to relative quantities and normalized to two reference genes, hypoxanthine phosphoribosyl transferase (*Hprt*) and 18S Ribosomal RNA (*Rn18s*), identified by geNorm software and shown to be stably expressed in tissue from MetS-Tg mice and controls, as previously described [26]. Gene expression data displayed consistently skewed distributions and were ln-transformed prior to statistical analysis.

### 2.7. Statistical Analysis 

Gender did not significantly alter the response to walnut diet in either genotype for any of the variables reported, as demonstrated in univariate comparisons, looking at Between-Subjects Effects for diet and gender and the diet-by-gender interaction (diet*gender) on each variable by ANOVA. Therefore, for subsequent comparisons, the genders were combined. To establish significant differences by genotype, diet, and the interaction between genotype and diet, between group comparisons were performed on indices of blood pressure, plasma biochemistry and levels of gene expression using ANOVA. Where significant associations were found, pairwise tests between significant variables were tested using *t*-tests. With 10 individuals in each genotype group (36 degrees of freedom), this study has sufficient power (80%) to detect moderate main effect sizes (>0.8 standard deviations) at *p* < 0.05. Results are expressed as means ± standard error of the mean. All statistical analyses were performed using SPSS version 22 (SPSS Inc., Chicago, IL, USA). A *p* value < 0.05 was taken to indicate statistical significance.

## 3. Results

**Figure 1:**
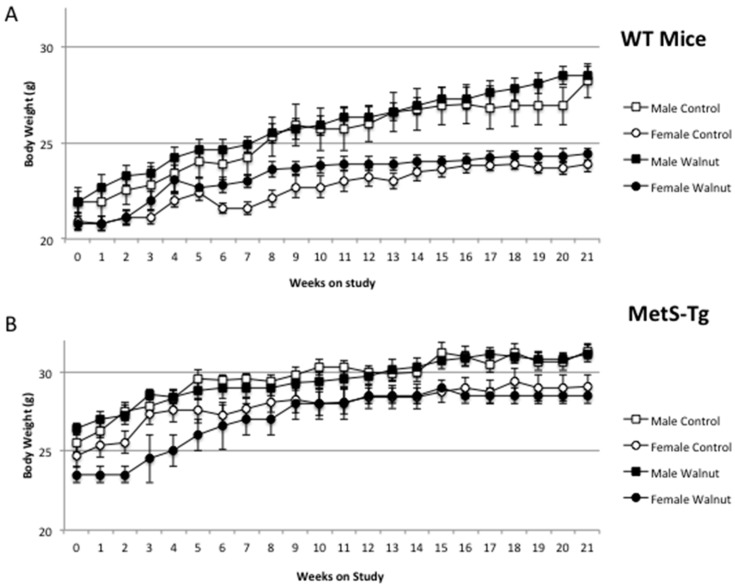
The time course body weight trajectories (mean ± sem) of wild-type (WT) mice (**A**) and MetS-Tg mice (**B**) over the 21 weeks of rodent standard diet (open symbols) or walnut supplemented diet (closed symbols), demonstrating that walnuts were not associated with additional weight gain.

Mean food consumption did not differ between WT and MetS-Tg mice on control or walnut diets (Table 2). The body weights of MetS-Tg mice were significantly higher than WT mice (effect of genotype, *p* < 0.001), evident both pre- and post-diet at 21 weeks (Figure 1), with male mice of both genotypes being significantly heavier than females (*p* < 0.001). However, there were no significant effects of walnut diet compared to standard rodent diet on body weight across the 21-week time course of the study (Table 1 and Figure 1). 

Walnuts were associated with a beneficial effect on oGTT glucose area under the curve (gAUC, Table 2), apparent only in the MetS-Tg mice. Pre-study, MetS-Tg mice had similar fasting plasma glucose levels compared to WT mice, but significantly higher gAUC (*p* = 0.006). Following the 21-week diets, the gAUC in WT mice was unchanged, while gAUC in MetS-Tg mice had decreased significantly with walnut supplementation (*p* = 0.003), to levels comparable with WT mice. In contrast, in MetS-Tg mice, the fasting plasma glucose levels were not changed from baseline, while WT mice had slightly higher fasting plasma glucose levels after the walnut diet (*p* = 0.022). 

Walnuts were associated with a major favourable effect on both mean arterial blood pressure (MAP) in the MetS-Tg mice in both WT and MetS-Tg mice (Table 2). There were significant effects of both genotype (*p* < 0.001) and diet (*p* < 0.001) on MAP, with MetS-Tg mice having a significantly higher MAP than WT mice pre-study. In WT mice, there was no significant change in MAP with diet. However, in MetS-Tg mice, the MAP continued to rise on the control diet, but MAP was reduced to levels comparable to WT mice after 21 weeks of walnut diet (*p* < 0.001 walnut versus control diet). 

At the end of the 21-day diet period, MetS-Tg mice exhibited significantly higher levels of plasma cholesterol (*p* < 0.001), triglycerides (*p* < 0.001), and creatinine (*p* = 0.001) compared to WT mice (Table 2). Compared with baseline, WT mice on the walnut diet showed a significant reduction in plasma cholesterol levels (*p* = 0.049) and no significant difference in plasma triglycerides. In contrast, plasma cholesterol and triglyceride levels, already significantly elevated in MetS-Tg versus WT mice, showed trends for further elevation after dietary walnuts; although neither increase reached statistical significance (cholesterol increased 1.5-fold, *p* = 0.138; triglycerides increased 2.25-fold, *p* = 0.061). The walnut diet was associated with no significant effects on plasma creatinine levels in either MetS-Tg (*p* = 0.165) or WT mice (*p* = 0.151).

**Figure 2:**
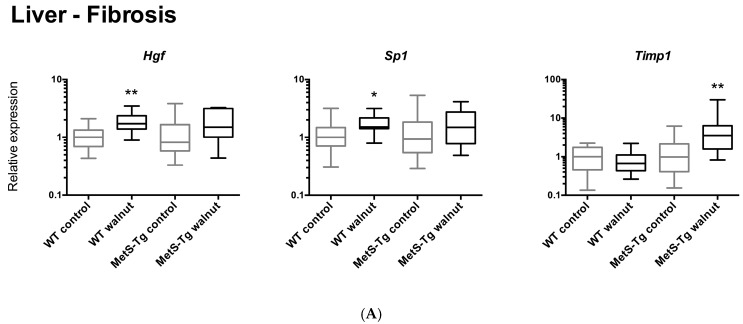

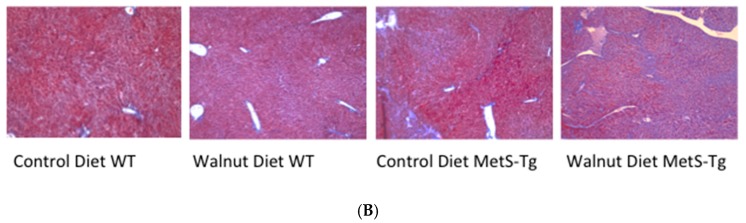
(**A**) Relative levels of gene expression in liver for genes associated with fibrosis (*Hgf*, *Sp1*, *Timp1*), as medians (Interquartile Ranges) with WT control expression set to 1, * *p* < 0.05, ** *p* < 0.005. (**B**) Representative sections of liver tissue stained with Masson Trichrome, indicating a gradient of interstitial fibrosis (blue), lowest in WT Control diet, and highest in MetS-Tg Walnut diet (10× magnification).

**Figure 3:**
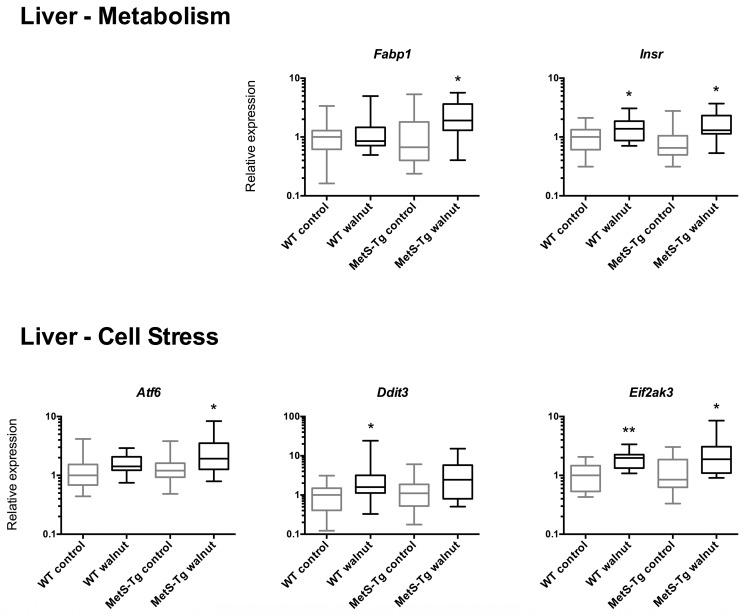

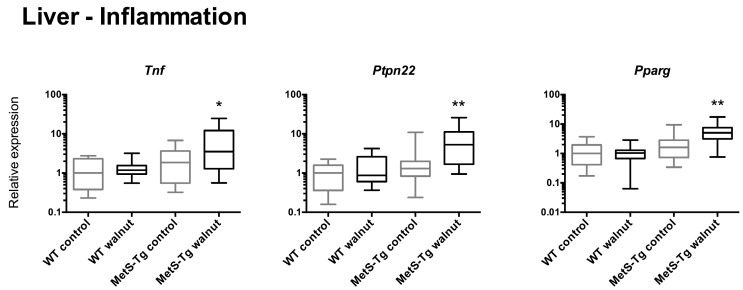
Relative levels of gene expression in liver for genes associated with metabolism (*Fabp1*, *Insr*, upper panel), cell stress (*Atf6*, *Ddit3*, *Eif2ak3*, central panel) and inflammation (*Tnf*, *Ptpn22*, *Pparg*, lowest panel), shown as medians (Interquartile Ranges) with WT control expression set to 1, * *p* < 0.05, ** *p* < 0.005.

**Figure 4:**
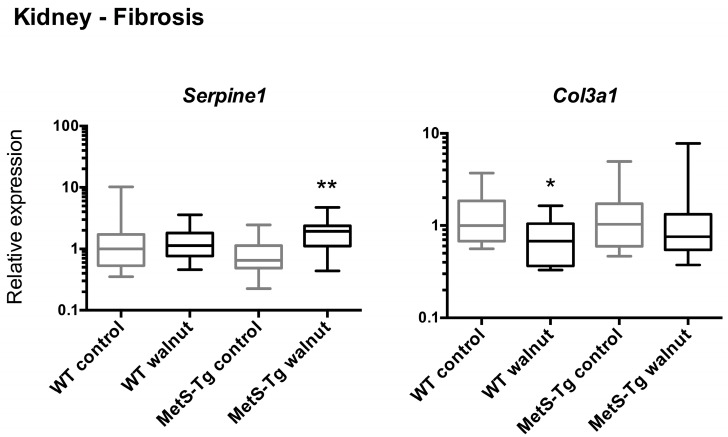
Relative levels of gene expression in kidney tissue for genes associated with fibrosis (*Serpine1*, *Col2a1*), shown as medians (Interquartile Ranges) with WT control expression set to 1, * *p* < 0.05, ** *p* < 0.005.

Relative expression levels of genes altered in association with walnut diet are shown in Figure 2, Figure 3 and Figure 4, and relative expression of all genes assayed are shown in Supplementary Table S2. In liver tissue, the majority of genes with altered expression demonstrated increased expression with the walnut diet in one or both of WT or MetS-Tg strains. This included genes associated with metabolism (*Fabp1*, *Insr*, Figure 2), genes associated with cell stress (*Atf6*, *Ddit3*, and *Eif2ak3*, Figure 3), genes associated with liver fibrosis (*Hgf*, *Sp1*, and *Timp1*, Figure 3) and genes associated with the inflammatory response (*Tnf*, *Ptpn22*, and *Pparg*, Figure 3). The decreased expression of *Slc2a4*, involved in glucose metabolism, in liver, especially in WT mice (Supplementary Table S2), did not reach statistical significance.

In kidney tissue, in response to walnut diet there was an increase in *Serpine1* (significant in MetS-Tg only, Figure 4) and a decrease in *Col3a1* expression (significant only in WT mice, Figure 4). Levels of *Mif* expression in both kidney and cardiac ventricle tissue were significantly lower in MetS-Tg than WT mice on both diets (Table S2), but not altered in association with walnut diet. In cardiac ventricle, there were no gross morphological changes or increased fibrosis evident, and of seven genes examined, none showed altered expression with walnut diet (Supplementary Table S2).

## 4. Discussion

We have investigated the nature of the putative beneficial effects of dietary walnut supplementation in a mouse model of MetS. This study documented a wide range of cardio-metabolic traits and related tissue gene expression associated with dietary supplementation with the equivalent to the daily walnut consumption (handful of walnuts) recommended for humans, adjusted for mouse body weight [22]. The walnut diet was isocaloric but had a slightly higher total polyunsaturated fat content compared with the standard diet (Supplementary Table S2). Overall, 21 weeks of walnut supplementation was associated with modest favourable effects on the cardiovascular and metabolic status of WT mice. However, in MetS-Tg mice, we observed evidence for a combination of clear beneficial (corrected glucose handling and reduction in blood pressure) and adverse (elevated blood cholesterol) effects. 

Our observation that walnut supplementation did not alter the body weight trajectories of either strain of mice and restored the elevated oGTT AUC of MetS-Tg mice to that of normal mice is consistent with previous reports. It has previously been shown that consumption of walnuts does not lead to increased body weight or BMI, may facilitate weight loss, (reviewed in [14,27]) and may have beneficial effects on insulin sensitivity, both in the healthy population and patients with metabolic disorders. The Nurses’ Health Study and Nurses’ Health Study II, a 10-year follow up of over 130,000 women indicated that regular walnut consumption was associated with a lower risk of DM2 [9], partly explained by a lower BMI. Moreover, walnut supplementation in overweight adults with DM2 was also associated with decreased fasting insulin [17], and with decreased fasting insulin and HOMA-IR in patients with the MetS [18]. The beneficial effect of walnuts in correcting glucose handling in MetS may offer a useful contribution to slowing the progression to diabetes.

In the current study, walnut supplementation was also associated with lowering the elevated blood pressure of MetS-Tg mice to that of normal WT mice. Most past studies that have measured blood pressure have reported no change with dietary walnuts [16,28]. However, several studies have reported improved endothelial function in association with walnuts, with increased flow-mediated dilation in overweight volunteers [29], in hyperlipidemic patients [30], and improved endothelial function in association with walnuts was confirmed in a systematic review [31]. There is evidence that endothelial and vascular function may be improved through increased production of nitric oxide from its precursor arginine, and through inhibition of endothelin-1 [27]. In addition, walnuts have been associated with reduced circulating adhesion molecules, such as vascular cell adhesion molecule-1 (VCAM-1) and E-Selectin [29,32], as well as reduced circulating inflammatory markers in humans and mice [18,33]. 

The current study found walnuts reduced total cholesterol in the WT mice, consistent with trials of walnut supplementation in healthy humans resulting in reduced total cholesterol, LDL, HDL [28], or total cholesterol, LDL, HDL, apolipoprotein B and triglycerides [34], or non-HDL cholesterol [10] summarized in reviews [6,35]. In the population-based Walnuts and Healthy Aging (WAHA) Study, walnut consumption was associated with decreased total cholesterol, LDL, and total: HDL-cholesterol ratios [19]. A study of ApoE-deficient mice fed a high-fat diet supplemented with walnuts for 8 weeks demonstrated reduced plasma total cholesterol and triglycerides, associated with reduced formation of atherosclerotic plaques in the aortic arch [36]. In combination, these factors are likely to contribute to protective effects of nuts against cardiovascular disease in the healthy general population. In the Physicians’ Health Study, there was an inverse association between nut consumption and CHD death, specifically sudden cardiac death, in over 21,000 participants followed for an average 17 years [11], which may reflect a combination of the beneficial effects described above. 

One explanation for the favourable cardio-metabolic actions of a diet rich in nuts and oils may be their effect on serum and tissue lipids. Nuts and oils are low in saturated fatty acids and high in unsaturated fatty acids, mainly monounsaturated fatty acids, omega-6 (linoleic) and omega-3 (linolenic) fatty acids [35]. Also, nuts are a source of dietary fiber; soluble fiber has a blood cholesterol-lowering effect [35]. Nuts also contain high amounts of arginine, the precursor of nitric oxide, a potent vasodilator that can inhibit platelet adhesion and aggregation and may contribute to the anti-atherogenic effect of nuts [37]. Walnuts differ from other nuts by having a particularly high content of α-linolenic acid, which may confer additional reduction in CHD risk [27]. In the PREDIMED trial, where adults at risk of CVD were randomized to a Mediterranean diet incorporating either olive oil or nuts for a median 5.9 years [12], a higher α-linolenic acid intake, principally from walnuts and olive oil, was associated with reduced all-cause mortality, although reductions in CVD mortality did not reach significance [13].

In contrast to the reduction of plasma lipids in response to walnut supplementation observed in WT mice, MetS-Tg mice demonstrated a strong trend for increased cholesterol and triglycerides with walnuts. This does not appear to result from the additional fat content of walnuts compounding the hyperlipidemic phenotype of these mice, since there is a plethora of papers reporting cholesterol-lowering effects of walnuts in hyperlipidemic patients [32,38,39,40,41]. Furthermore, in the ApoE-deficient mouse (one of the parent strains of our MetS mouse model), which displays high cholesterol and a predisposition for atherosclerosis but not a pre-diabetic phenotype, walnuts added to a high-fat diet lowered plasma cholesterol, triglycerides and prothrombin compared to high-fat diet alone [36]. Our finding that walnuts fail to exhibit their lipid-lowering effect in the MetS-Tg mouse model of metabolic dysfunction is supported by the few studies of walnuts in MetS or pre-diabetic humans. When MetS patients were randomized to a healthy diet with or without walnut supplementation, a reduction of LDL was seen in the control but not the nut diet arm of this trial [18]. Similarly, in a study in which DM2 patients were randomized to low-fat dietary advice with or without walnuts for over 1 year, walnuts reduced HDL but there was no additional reduction in other lipid fractions in the walnut group compared with the control group [17]. One study of adults classed as at-risk for diabetes randomly assigned to walnut-included diet versus a walnut-excluded diet for 6 months [16], reported both the walnut-included and control diets demonstrated a similar reduction in total and low density lipoprotein (LDL) cholesterol from baseline. Further, in a weight loss intervention in overweight or obese women, a walnut-rich diet increased HDL, but reduced LDL in insulin-sensitive women only, not in those classified as insulin resistant [15]. In contrast, a recent randomized control trial of 3 months supplementation with walnut oil in hyperlipidemic DM2 patients found significantly reduced total cholesterol, LDL, and triglycerides [20], suggesting that walnut oil consumption may differ from whole walnuts in the effects on lipid profiles. Reasons for the divergent lipid response to walnuts in MetS patients compared to healthy controls are discussed by Casas-Agustench and co-workers [18], who suggest that high cholesterol synthesis, reduced intestinal cholesterol absorption and enhanced cholesterol flux through the liver resulting in LDL receptor down-regulation, may render insulin-resistant individuals less responsive to cholesterol-lowering diets. 

An unexpected finding of the current study was the elevated levels of hepatic gene expression of cell signalling genes associated with metabolism, cell stress, inflammation and fibrosis in association with the walnut diet, changing in concert in both WT and MetS-Tg mice. Moreover, increased histological staining for fibrosis in liver sections corroborated these expression changes. In contrast, we observed discordant expression of pro-fibrotic signals in kidney tissue, with up-regulation of *Serpine1* and down-regulation of *Col3a1* in MetS-Tg and WT mice, respectively. Genes selected for measurement in liver were those that had demonstrated altered expression previously in association with a fast-food diet (high saturated fat, cholesterol and fructose) in a mouse model of non-alcoholic fatty liver disease [42], with significantly elevated expression of eight genes in common with the current study. However, these findings contrast with other findings of reduced inflammatory cytokines in adipose tissue of mice fed a high-fat diet [43], and in serum of walnut-fed mice accompanied by altered gene expression in unspecified tissues [33]. In MetS patients, walnuts were reported to decrease circulating levels of the pro-inflammatory cytokines, monocyte chemotactic protein-1 (MCP-1), IL-6 and IL-18, but not C-reactive protein (CRP) or PAI-1 [18]. The gene expression pattern observed in the current study and in a fast-food mouse model [42], contrasts to the lack of change in liver gene expression associated with a high-fat diet in the latter study by Charleton et al. Compared to standard diet, the fast-food diet elicited significantly higher liver gene expression levels of anti-smooth muscle actin (*ASMA*), *TGFβ1*, *Col1α_1_*, *Lum*, *Timp1, Hgf*, *Sp1*, *Tnf*, *Spp1*, and *Ddit3* while *Fabp1* and *Eif2ak3* displayed lower expression [42]. Why the walnut diet in the current study and the fast-food diet would be associated with similar patterns of up-regulation of pro-inflammatory, pro-fibrotic genes in liver is not obvious; however, the fast-food diet elicited a MetS-like phenotype, with a doubling of body weight, an almost 4-fold rise in homeostatic model assessment-insulin resistance (HOMA-IR), a 5-fold rise in serum cholesterol and doubling of serum aspartate aminotransferase (AST, a marker of liver injury). In combination, these data suggest that fatty foods, even foods containing mainly unsaturated fatty acids such as walnuts, alter liver cell signalling pathways, evident even in healthy individuals.

Conducting dietary studies in mice confers several advantages over human dietary studies. Firstly, inbred mouse disease models and control WT strains do not carry the genetic and environmental diversity that human patients and healthy controls do, with disparities in response arising from diverse medical histories, medications and lifestyle factors. Secondly, the intake of the nutrient of interest can be more closely controlled and monitored in mice than in humans. Finally, mice are blinded to the dietary intervention they are randomized to, while human studies frequently observe changes from baseline in the control arm as well as the intervention arm of the study, likely due to the Hawthorne effect, or the awareness of being observed. This is apparent in the walnut literature, where several studies have noted that both walnut-supplemented and control groups showed significant improvements from baseline measurements [17,39].

Limitations of our study include that levels of other cholesterol fractions, such as HDL-cholesterol, were not measured in this study due to the limited plasma volume available from mice. The diet was maintained for only 21 weeks and the WT may have shown detrimental effect of walnuts on lipids or metabolic indices if the diet had been continued for longer. The walnut versus standard diet were intentionally not balanced for fat, as we set out to model the effects of an individual adding a handful of walnuts to their usual daily diet. Thus the estimated content of beneficial polyunsaturated fats were slightly higher in the walnut diet compared to the standard diet, although we cannot determine which of these contributed to the observed physiological responses. Also, we acknowledge that while the liver gene expression changes are intriguing, the regulation of the processes of tissue fibrosis, inflammation and cell stress are more complex than mRNA expression levels can convey. Other mechanisms may regulate these pathways, such as alternative splicing, whereby mRNA splice variants have differential effects; post-translational modifications (e.g., phosphorylation, glycosylation); or epigenetic regulation of the translation of the final protein (DNA methylation, histone modification or microRNAs). Further research will be needed to clarify the functional implications of gene expression changes observed in the current study. 

## 5. Conclusions 

The current study in mice suggests the response to walnuts is influenced by the metabolic status of the individual. Walnut supplementation for 21 weeks was associated with small favourable or neutral effects on the cardiovascular and metabolic status of healthy WT mice, but in MetS-Tg mice walnuts were associated with profound benefits for insulin sensitivity and blood pressure but adverse effects on cholesterol and triglycerides. Moreover, we demonstrated up-regulated expression of pro-fibrotic and pro-inflammatory genes associated with the walnut diet in the livers of both strains of mice. This suggests that walnuts may make a useful contribution to slowing the progression to diabetes, providing lipid profiles are closely monitored. Further research investigating the effects of more prolonged walnut supplementation in mice is required, to evaluate whether the observed changes in hepatic gene expression are deleterious in the long term. Ultimately, the benefits of walnut supplementation in MetS will need to be established in a long-term randomized control trial in a MetS patient cohort, examining clinical endpoints such as fatal and non-fatal CVD and all-cause mortality.

## Figures and Tables

**Table 1:** Description of Genes of Interest and Reference Genes.

Gene Symbol	Gene Name	Taqman Assay ID	Gene Function
Genes of Interest			
*Trp53 (p53)*	Transformation related protein 53	Mm01731290_g1	Apoptosis
*Myh7b*	Myosin, heavy chain 7B, cardiac muscle, beta	Mm01249941_m1	Cardiac fibrosis
*Atf6*	Activating transcription factor 6	Mm01295317_m1	Cellular Stress
*Bbc3 (PUMA)*	BCL2 binding component 3	Mm00519268_m1	Cellular Stress
*Ddit3 (CHOP)*	DNA-damage inducible transcript 3	Mm01135937_g1	Cellular Stress
*Eif2ak3 (PERK)*	Eukaryotic translation initiation factor 2 alpha kinase 3	Mm00438700_m1	Cellular Stress
*Fabp1*	Fatty acid binding protein 1, liver	Mm00444340_m1	Fatty acid trafficking
*Fabp4*	Fatty acid binding protein 4, adipocyte	Mm01295675_g1	Fatty acid trafficking
*Col1a1*	Collagen, type I, alpha 1	Mm00801666_g1	Fibrosis
*Col3a1*	Collagen, type III, alpha 1	Mm01254476_m1	Fibrosis
*Hgf*	Hepatocyte growth factor	Mm01135193_m1	Fibrosis
*Lum*	Lumican	Mm01248292_m1	Fibrosis
*Serpine1 (PAI-1)*	Serine peptidase inhibitor, clade E, member 1	Mm00435860_m1	Fibrosis
*Sp1*	Trans-acting transcription factor 1	Mm00489039_m1	Fibrosis
*Tgfb1*	Transforming growth factor, beta 1	Mm01178820_m1	Fibrosis
*Timp1*	Tissue inhibitor of metalloproteinase 1	Mm00441818_m1	Fibrosis
*Insr*	Insulin receptor	Mm01211875_m1	Glucose metabolism
*Slc2a4 (GLUT 4)*	Solute carrier family 2 (facilitated glucose transporter), member 4	Mm01245502_m1	Glucose metabolism
*Havcr1 (KIM-1)*	Hepatitis A virus cellular receptor 1	Mm00506686_m1	Inflammation
*Il6*	Interleukin 6	Mm00446190_m1	Inflammation
*Mif*	Macrophage migration inhibitory factor	Mm03938638_s1	Inflammation
*Pparg*	Peroxisome proliferator activated receptor gamma	Mm01184322_m1	Inflammation
*Ptpn22*	Protein tyrosine phosphatase, non-receptor type 22 (lymphoid)	Mm00501246_m1	Inflammation
*Sod1*	Superoxide dismutase 1, soluble	Mm01344233_g1	Inflammation
*Spp1 (Osteopontin)*	Osteopontin/secreted phosphoprotein 1	Mm00436767_m1	Inflammation

**Table 2:** Physiological and blood biochemistry data pre- and post-study diets (means ± SEM).

	Wild-Type			MetS-Tg		
	Pre-Study	Control Diet	Walnut Diet	Pre-Study	Control Diet	Walnut Diet
Food Consumption (g/24 h)	-	3.7 ± 0.6	3.6 ± 0.1	-	3.6 ± 0.3	3.6 ± 0.1
Body weight females (g)	20.9 ± 0.4	23.9 ± 0.4	24.4 ± 0.3	24.7 ± 0.7 ***	29.1 ± 0.8 ***	28.5 ± 0.5 ***
Body weight males (g)	21.9 ± 0.8	28.2 ± 9	28.5 ± 0.5	25.6 ± 0.7 ***	31.3 ± 0.5 ***	31.2 ± 0.6 ***
Fasting Glucose (mmol/L)	6.6 ± 0.2	5.8 ± 0.2	7.1 ± 0.2 ^##^	6.7 ± 0.3	6.6 ± 0.2	6.7 ± 0.4
gAUC	1154 ± 57	1221 ± 51	1140 ± 51	1409 ± 69 *	1455 ± 54	1146 ± 91 ^#^
MAP (mmHg)	71.4 ± 1.2	74.0 ± 0.8	76.0 ± 1.7	83.2 ± 2.9 ***	100.6 ± 1.9	73.2 ± 1.8 ^###^
Plasma Cholesterol (mmol/L)	-	2.12 ± 0.13	1.60 ± 0.18 ^#^	-	4.73 ± 0.08 ***	7.03 ± 1.99
Plasma Triglycerides (mmol/L)	-	0.93 ± 0.2	0.88 ± 0.1	-	2.4 ± 0.5 ***	5.4 ± 1.6
Plasma Creatinine (μmol/L)	-	100 ± 20	160 ± 30	-	690 ± 190 ***	300 ± 130

* *p* < 0.05, *** *p* < 0.001 in MetS-Tg vs. WT mice ^#^ *p* < 0.05, ^##^ *p* < 0.005, ^###^ *p* < 0.001 walnut vs. control diet in the same strain of mice.

## Supplementary Materials

The following are available online at www.mdpi.com/2072-6643/9/7/722/s1, Table S1: Control Diet Composition, Table S2: Relative Expression Data for All Genes Analyzed.
