# Supplementary material for: Metabolic and Blood Pressure Effects of Walnut Supplementation in a Mouse Model of the Metabolic Syndrome
Source: Nutrients. 2017 Jul 7;9(7):722. doi: 10.3390/nu9070722 (PMC5537837; doi:10.3390/nu9070722)
Supplement: Supplementary file 1 [file nutrients-09-00722-s001.zip › nutrients-206912-supplementary final.pdf]

Table S1: Control Diet Composition as provided by the manufacturer (Specialty Feeds, Glen Forrest, Western Australia) and estimated key macronutrients in the walnut diet (0.3% walnuts). Addition of walnuts did not change Vitamin content from standard diet. Ingredients: Wheat, barley, Lupins, Soya meal, fish meal, Mixed vegetable oils, Canola oil, Salt, Calcium carbonate, Dicalcium phosphate, Magnesium oxide, and a Vitamin and trace mineral premix.

**Nutritional Parameter**	**Standard Diet**	**Standard Diet including Walnuts**
Protein	20.00% wt	19.00%
Total Fat	4.80% wt	4.98%
Total Carbohydrate	59.40% wt	59.26%
Digestible Energy	14.00 MJ/kg	14.04 MJ/kg
L-Arginine	No data	19.4 mg/kg
**Calculated Fatty Acid Composition**		
Myristic Acid 14:0	0.03%	0.03%
Palmitic Acid 16:0	0.50%	0.52%
Stearic Acid 18:0	0.14%	0.15%
Palmitoleic Acid 16:1	0.01%	0.01%
Oleic Acid 18:1	1.90%	1.96%
Gadoleic Acid 20:1	0.03%	0.03%
Linoleic Acid 18:2 n6	1.30%	1.47%
α Linolenic Acid 18:3 n3	0.30%	0.33%
Arachadonic Acid 20:4 n6	0.01%	0.01%
EPA 20:5 n3	0.02%	No data
DHA 22:6 n3	0.05%	No data
Total n3	0.37%	0.40%
Total n6	1.31%	1.42%
Total Mono Unsaturated Fats	2.00%	2.02%
Total Polyunsaturated Fats	1.77%	1.91%
Total Saturated Fats	0.74%	0.76%
**Calculated Total Vitamins**		
Vitamin A (Retinol)	19500 IU/kg	Unchanged
Vitamin D (Cholecalciferol)	2000 IU/kg	Unchanged
Vitamin E (a Tocopherol acetate)	110 mg/kg	Unchanged
Vitamin K (Menadione)	20 mg/kg	Unchanged
Vitamin C (Ascorbic acid)	No data	Unchanged
Vitamin B1 (Thiamine)	80 mg/kg	Unchanged
Vitamin B2 (Riboflavin)	30 mg/kg	Unchanged
Niacin (Nicotinic acid)	145 mg/kg	Unchanged
Vitamin B6 (Pryridoxine)	28 mg/kg	Unchanged
Pantothenic Acid	60 mg/kg	Unchanged
Biotin	410 ug/kg	Unchanged
Folic Acid	5 mg/kg	Unchanged
Inositol	No data	Unchanged
Vitamin B12 (Cyancobalamin)	150 ug/kg	Unchanged
Choline	1600 mg/kg	Unchanged

Table S2: Relative Expression Data for All Genes Analyzed
Expressed as medians (Interquartile Ranges) relative to WT Control Diet (set to 1). Human homologs or alternative gene names are given in parentheses after mouse gene name.

**Genotype**<br>**Diet**	**WT**<br>**Control**	**WT**<br>**Walnut**	**MetS-Tg**<br>**Control**	**MetS-Tg**<br>**Walnut**	***P Genotype***	***P Diet***	***P Geno*diet***
**Liver**							
Atf6 (ATF6)	1.00 (0.69-1.53)	1.42 (1.22-2.07)	1.20 (0.93-1.61)	1.93 (1.26-3.53)		*0.005*	
Ddit3 (CHOP)	1.00 (0.41-1.50)	1.59 (1.13-3.17)	1.11 (0.52-1.87)	2.45 (0.80-5.76)		*0.002*	
Eif2ak3 (PERK)	1.00 (0.53-1.46)	1.98 (1.33-2.26)	0.84 (0.63-1.86)	1.88 (1.09-3.07)		*<0.001*	
Col1a1 (COL1A1)	1.00 (0.68-1.45)	0.54 (0.35-0.99)	0.97 (0.40-1.50)	2.61 (1.26-4.30)	*0.004*		*0.002*
Hgf (HGF)	1.00 (0.69-1.34)	1.72 (1.39-2.35)	0.82 (0.58-1.66)	1.50 (1.01-3.15)		*0.001*	
Lum (LUM, Lumican)	1.00 (0.34-2.39)	0.83 (0.38-1.59)	0.58 (0.32-1.51)	1.60 (0.59-3.85)			
Spp1 (SPP1, Osteopontin)	1.00 (0.54-1.83)	0.95 (0.75-1.38)	1.27 (0.34-2.41)	1.63 (0.58-4.67)			
Sp1 (SP1)	1.00 (0.71-1.48)	1.51 (1.42-2.16)	0.94 (0.55-1.84)	1.50 (0.78-2.74)		*0.011*	
Tgfb (TGFB1)	1.00 (0.47-1.96)	0.65 (0.49-1.63)	1.51 (0.49-2.22)	2.00 (1.20-4.80)	*0.007*		
Timp1 (TIMP1)	1.00 (0.46-1.74)	0.67 (0.43-1.11)	0.99 (0.41-2.14)	3.54 (1.58-6.37)	*<0.001*	*0.018*	*0.003*
Tnf (TNF)	1.00 (0.38-2.31)	1.18 (0.94-1.56)	1.87 (0.55-3.64)	3.52 (1.28-12.15)	*0.002*	*0.021*	
Sod1 (SOD1, ALS)	1.00 (0.67-1.40)	0.98 (0.67-1.70)	1.00 (0.49-1.60)	2.43 (0.60-4.21)			
Bbc3 (PUM, BBC3)	1.00 (0.52-1.64)	0.87 (0.51-1.52)	0.64 (0.41-1.27)	1.46 (0.51-2.25)			
Fabp1 (FABP1)	1.00 (0.62-1.29)	0.85 (0.71-1.46)	0.67 (0.40-1.80)	1.91 (1.30-3.64)		*0.028*	
Ptpn22 (PTPN22)	1.00 (0.36-1.58)	0.87 (0.60-2.61)	1.30 (0.83-1.99)	5.29 (1.67-11.21)	*<0.001*	*<0.001*	
Pparg (PPARG)	1.00 (0.42-1.94)	1.03 (0.68-1.28)	1.61 (0.73-2.84)	5.02 (3.12-7.53)	*<0.001*	*0.029*	*0.016*
Il6 (IL6)	1.00 (0.55-2.01)	1.35 (0.71-2.01)	0.92 (0.49-2.25)	1.68 (0.81-5.62)			
Insr (INSR)	1.00 (0.61-1.33)	1.38 (0.87-1.86)	0.65 (0.50-1.05)	1.30 (1.13-2.31)		*<0.001*	
Slc2a4 (Glut4)	1.00 (0.49-1.38)	0.46 (0.21-0.72)	0.92 (0.09-4.99)	0.91 (0.31-1.43)			
**Kidney**							
Cst3 (CST3, cystatin C)	1.00 (0.61-1.97)	0.74 (0.63-1.12)	1.14 (0.56-1.76)	0.84 (0.49-1.73)			
Havcr1 (HAVCR1, Kim1)	1.00 (0.65-2.34)	0.45 (0.14-1.10)	0.20 (0.06-0.37)	0.07 (0.03-0.68)	*0.001*		

Serpine 1 (SERPINE1, Pai1)	1.00 (0.53-1.73)	1.13 (0.77-1.82)	0.65 (0.49-1.13)	1.93 (1.11-2.37)		*0.028*	*0.042*
Tgfb (TGFB1)	1.00 (0.55-1.66)	0.82 (0.60-1.16)	0.90 (0.64-1.81)	0.88 (0.67-1.85)			
Col3a (COL3a)	1.00 (0.68-1.85)	0.68 (0.37-1.05)	1.03 (0.60-1.73)	0.76 (0.55-1.33)		*0.040*	
Mif (MIF)	1.00 (0.76-1.69)	1.05 (0.53-1.55)	0.50 (0.31-1.18)	0.80 (0.42-1.15)	*0.026*		
Trp53 (TP53, p53)	1.00 (0.72-2.08)	0.91 (0.66-2.15)	1.16 (0.71-2.84)	1.94 (1.16-2.55)			
**Cardiac Ventricle**							
Col1a1 (COL1A1)	1.00 (0.52-1.91)	0.98 (0.32-2.11)	0.79 (0.36-1.50)	0.62 (0.44-0.85)			
Tgfb (TGFB1)	1.00 (0.68-3.75)	1.62 (0.59-3.91)	0.80 (0.34-2.68)	0.98 (0.35-1.11)			
Col3a1 (COL3a1)	1.00 (0.70-1.63)	1.40 (0.43-2.08)	0.84 (0.34-1.71)	0.59 (0.38-0.88)			
Fabp4 (FABP4)	1.00 (0.60-1.56)	1.17 (0.37-2.87)	0.59 (0.26-1.64)	0.78 (0.34-1.43)			
Mif (MIF)	1.00 (0.28-4.14)	0.60 (0.15-4.47)	0.38 (0.06-0.78)	0.26 (0.14-0.85)	*0.022*		
Myh7b (MYH7B)	1.00 (0.59-3.10)	2.07 (0.61-2.40)	0.56 (0.35-2.01)	0.84 (0.32-1.61)			
Trp53 (TP53, p53)	1.00 (0.52-1.87)	1.56 (0.46-2.54)	0.93 (0.50-1.55)	0.75 (0.52-1.08)			
